# The impact of sleep quality on emotion regulation difficulties in adolescents: a chained mediation model involving daytime dysfunction, social exclusion, and self-control

**DOI:** 10.1186/s12889-024-19400-1

**Published:** 2024-07-11

**Authors:** Wenjuan Wang, Yuqiong Zhu, Hang Yu, Chengcong Wu, Tiancheng Li, Chenguang Ji, Yulian Jiang, Dongyan Ding

**Affiliations:** 1 Bengbu Medical University, Bengbu, Anhui 233030 China; 2 Anhui Normal University, Wuhu, Anhui 241000 China

**Keywords:** Adolescents, Sleep quality, Daytime dysfunction, Social exclusion, Self-control, Emotion regulation, Chained mediation effects

## Abstract

**Objective:**

Previous studies have revealed associations between sleep quality and mental health, yet the comprehensive role of sleep quality, daytime dysfunction, social exclusion, and self-control in difficulties with emotion regulation remains unclear. This study aimed to elucidate how sleep quality affects emotion regulation difficulties among middle school students through pathways involving daytime dysfunction, social exclusion, and self-control, thereby providing a more comprehensive theoretical basis for mental health interventions.

**Methods:**

Utilizing the pittsburgh sleep quality index, the adolescent social exclusion scale, the brief self-control scale, and emotion regulation scale-short form, we assessed 1067 students randomly selected from four middle schools from October to November 2023. After the removal of extreme values (those exceeding 3 standard deviations), 806 students were retained for data analysis.

**Results:**

Our findings indicate that poor sleep quality significantly contributes to increased daytime dysfunction(*β* = 0.86, *SE* = 0.07, *p* < .001), which in turn affects social exclusion(*β* = 0.60, *SE* = 0.16, *p* < 0 0.001), self-control abilities(*β* = 1.27, *SE* = 0.16, *p* < .001) and emotion regulation difficulties(*β* = 1.56, *SE* = 0.30, *p* < .001). Social exclusion mediates the relationship between sleep quality and emotion regulation difficulties(Estimate = 0.11, *SE* = 0.04, 95% *CI* [0.04, 0.20] ).

**Conclusion:**

The aim of this study is to provide new insights into the development of effective intervention measures to improve sleep and mental health in adolescents.

## Introduction

Emotion regulation, the process through which individuals influence their own emotional experiences and expressions, is particularly critical for the psychological health and social development of middle school students [1]. Adolescence represents a pivotal period marked by rapid physiological and psychological transformations that intensify challenges associated with emotion regulation [2]. Evidence suggests that deficiencies in emotion regulation capabilities are linked to a spectrum of mental health issues, including anxiety, depression, and behavioral problems [3]. Furthermore, recent studies indicate that over 40% of middle school students report difficulties in emotion regulation, which significantly correlates with future mental health problems [4]. However, schools often prioritize academic achievement, overlooking the importance of developing emotion regulation skills [5].

The quality of sleep significantly impacts emotion regulation. Insufficient sleep has been firmly established as both a risk factor and a maintaining element for mental health issues, with emotion regulation serving as a crucial intermediary [6]. Research has illuminated a profound interconnection between sleep and emotion regulation, highlighting that high-quality sleep is fundamental to emotional stability and overall psychological well-being [7, 8]. Close associations have been documented between sleep quality and difficulties in emotion regulation [7]. Adequate sleep is considered crucial for emotional stability and mental well-being [8]. The influence of sleep on emotion regulation manifests primarily in two aspects: emotional recognition and emotional response. For instance, sleep deprivation can impair the functioning of brain regions responsible for processing emotional information, such as the amygdala and prefrontal cortex, thus reducing the accuracy of emotional recognition [9]. Moreover, poor sleep quality can heighten negative emotional responses, potentially leading to an increase in symptoms of anxiety and depression [8, 10]. Research has found that adolescents with insufficient sleep are more prone to emotional volatility and impulsive behaviors [11]. Additionally, the impact of sleep on emotion regulation can also be understood from the perspectives of stress response and recovery; sleep deprivation can lead to an exaggerated response to stress and elevated cortisol levels, thereby affecting emotional stability [12]. Chronic sleep insufficiency may result in a state of chronic stress, further exacerbating difficulties in emotion regulation [13].

Poor sleep quality has a profound and multifaceted impact on adolescents, extending far beyond mere tiredness to encompass a range of cognitive, emotional, and social dysfunctions. Daytime dysfunction significantly compromises adolescents’ physical health and has far-reaching implications for their psychological well-being and social life [14], while poor sleep quality has a direct impact on daytime dysfunction, leading to cognitive decline manifesting as diminished attention, impaired memory, and compromised decision-making abilities [15]. The Cognitive Resource Theory posits that individuals rely on limited cognitive resources when engaging in cognitive tasks [16], thus insufficient sleep can reduce the cognitive resources available for effectively social interactions, self-control, and regulating emotions.

According to cognitive resource theory, poor social perception and cognition resulting from sleep deprivation make it difficult for individuals to accurately interpret social cues and understand others’ emotions [17]. This can result in social awkwardness or inappropriate social behaviors, leading to social exclusion [17]. Adolescents facing sleep-related issues are particularly susceptible to experiencing heightened levels of anxiety and discomfort in social settings, potentially leading to feelings of alienation and social exclusion [18]. This sense of isolation can be further exacerbated by the negative impact of sleep deprivation on academic performance, which in turn can diminish social standing and strain peer relationships [19]. Moreover, the detrimental effects of sleep deprivation extend to self-regulatory processes, particularly impairing the functionality of the prefrontal cortex, a brain region integral to self-control [20, 21]. The compromised self-control can manifest in increased impulsivity, difficulty in planning, and challenges in executing complex tasks, further complicating adolescents’ emotion regulation. Adolescence is a critical period of brain development maturation, and insufficient sleep may have a negative impact on the prefrontal cortex, which in turn affects an individual’s self-control ability [22]. Existing studies have explored the association between sleep and self-control using various methodologies. For instance, Fava et al. employed a longitudinal design to examine the impact of sleep patterns on self-regulatory behaviors in adolescents, highlighting the persistent effects of sleep deprivation on prefrontal cortex development and subsequent self-control difficulties [23]. Similarly, Lau et al. utilized a combination of self-reported sleep measures and neuroimaging techniques to investigate the neural mechanisms underlying the sleep-self-control relationship, finding that poor sleep quality is associated with decreased activation in prefrontal cortical regions during tasks requiring self-regulation [24].

The Social Pain Overlap Theory (SPOT) posits that the brain regions involved in processing physical pain overlap significantly with those involved in processing social pain, such as feelings of rejection or exclusion. According to Eisenberger and Lieberman [25], this theory suggests that social exclusion triggers a response in the brain similar to that of physical pain, which can have profound effects on emotional and cognitive functioning. Some empirical findings suggested that social exclusion invokes negative emotions such as loneliness, depression, and anxiety, which can undermine self-control and emotion regulation capacities [26]. Grappling with feelings of rejection may deplete the cognitive resources necessary for effective self-control, leaving individuals less equipped to manage their emotional responses adaptively. Some research found that individuals who experience social exclusion may be more inclined to engage in impulsive behaviors, such as overeating, risky activities, or aggressive actions, as a coping mechanism to counteract feelings of rejection [26, 27]. These impulsive behaviors signify a lapse in self-control. The depletion of cognitive resources, coupled with an increase in behavioral impulsivity, further exacerbates the challenges faced by adolescents in maintaining self-control and regulating emotions effectively [27].

In summary, the intricate interplay among sleep quality, daytime dysfunction, social exclusion, and self-control collectively impacts adolescents’ emotion regulation capabilities. Accordingly, this study proposes that the relationship between sleep quality and emotion regulation difficulties is mediated by three variables: daytime dysfunction, social exclusion, and self-control. These five variables together form a chained mediation effect model that illustrates the influence of sleep quality on emotion regulation difficulties.

To date, no research has comprehensively examined the interrelationships among these five variables. Most existing studies tend to focus on one or two of these variables in isolation, failing to fully elucidate their interplay and combined effects. By integrating multiple related variables, this model aims to provide a more comprehensive perspective for understanding the factors affecting emotion regulation and to unveil the potential mechanisms linking sleep quality with emotion regulation difficulties.

## Methods

### Participants

This study recruited students from four schools, including two junior high schools and two senior high schools, located in the local area. The schools were selected through a random sampling method to ensure representativeness of the sample. A total of 1067 students participated in the study. After the removal of extreme values (those exceeding 3 standard deviations), 806 questionnaires were retained for subsequent data analysis. The sample comprised 51.5% male students, with 27% in the 7th grade, 23.3% in the 8th grade, 26.2% in the 10th grade, and 23.5% in the 11th grade. The average age of the students was 14.35 years, with a standard deviation of 1.66. Additionally, 52.6% of the students had urban household registrations, and 55% reported bedtime later than 23:00. There was no significant difference in school start times across the four schools we surveyed, with grades 7 and 8 end time earlier than grades 10 and 11.

Univariate analysis of variance (ANOVA) has revealed marked age-related disparities between junior high school grades (7th and 8th grades) and senior high school grades (10th and 11th grades) across a range of variables. Students in the junior high grades exhibit significantly superior sleep quality and self-control compared to their senior high counterparts, accompanied by reduced daytime dysfunction and emotional dysregulation. Notably, no significant variation exists among the grades concerning social exclusion. Specifically, both 7th and 8th graders demonstrate significantly higher sleep quality than 10th and 11th graders, while also encountering fewer issues related to daytime functional impairment and emotional dysregulation, and exhibiting enhanced self-regulatory capabilities. Intriguingly, no statistically significant differences are observed between the 7th and 8th grades across these measured parameters. Similarly, the 10th and 11th grades do not differ significantly from each other across the examined variables, pointing to a similar trend of consistency within the senior high context. Independent samples t-tests revealed no statistically significant differences between genders concerning sleep quality or experiences of social exclusion. However, significant differences emerged in terms of daytime dysfunction, self-control, and emotion regulation difficulties, where males exhibited higher functioning, better self-control, and less difficulty in emotion regulation compared to females. Given the significant differences observed across demographic variables such as age and class, we will incorporate these variables as covariates in our analysis.

**Fig. 1:**
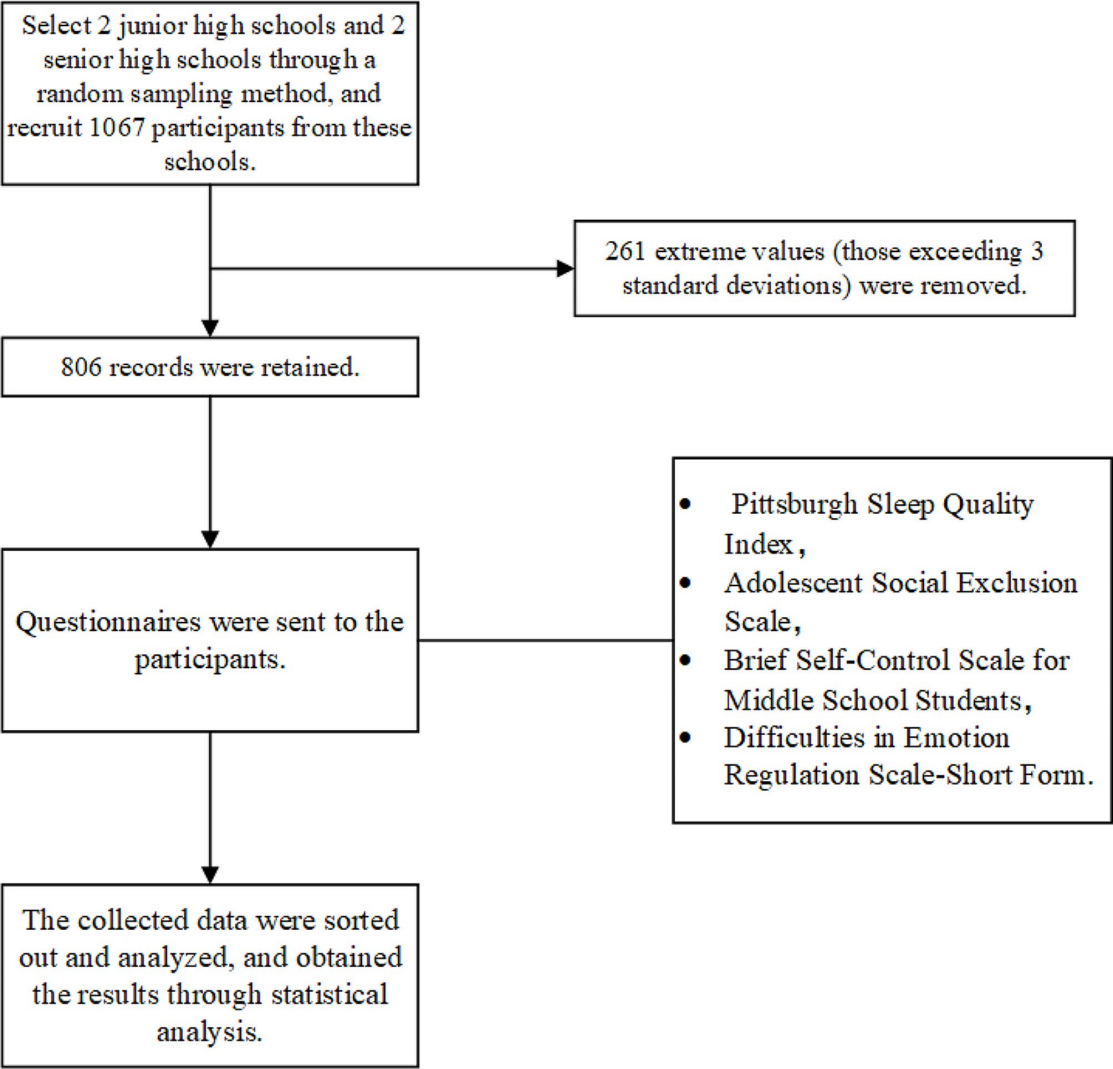
The flowchart of the study

Prior to the commencement of the study, we provided detailed information to all participants and their guardians regarding the research objectives, procedures, and privacy protection measures, obtaining their written informed consent. Throughout the study, we strictly adhered to relevant ethical standards to ensure the confidentiality of participants’ personal information and responses. The flowchart is shown in Fig. 1.

### Measures

Students are required to report their demographic information, including gender, grade level, and their late sleeping habits. Regarding late sleeping habits, a cutoff time of 23:00 is adopted, where going to bed later than 23:00 is classified as staying up late, whereas retiring at or before 23:00 is considered as not engaging in late-night activities [28].

#### Pittsburgh sleep quality index

This study used the Pittsburgh Sleep Quality Index (PSQI) to evaluate sleep quality and daytime dysfunction. PSQI is a widely used tool that consists of seven components, covering various aspects of subjective sleep quality and daytime dysfunction [29]. In this research, only the components pertaining to sleep quality and daytime dysfunction were selected. Sleep quality is evaluated through a project, with higher scores indicating poorer self-reported sleep quality. Two items were evaluated for daytime functional impairment, and the higher the score, the more severe the functional impairment. Both components utilized a Likert 4-point scoring system. In our study, the internal consistency reliability was computed based on the three specific items selected from the PSQI and the Cronbach’s alpha coefficient in this study was 0.79, indicating good reliability.

#### Adolescent social exclusion scale

Using the Adolescent Social Exclusion Scale to assess the degree of social exclusion. This Chinese version of this scale consists of 11 items, divided into two dimensions: ignore and reject. Use the Likert 5-point scoring system to evaluate based on a score range from 1 (never) to 5 (always) [30]. The total score is calculated by adding up the scores of all items, and the higher the score, the higher the degree of social exclusion. In our study, the scale demonstrated good reliability with a Cronbach’s alpha coefficient of 0.87.

#### Brief self-control scale for middle school students

Self-control capability was assessed using the Brief Self-Control Scale for Middle School Students [31]. The scale’s reliability and validity have been confirmed in previous research, making it suitable for evaluating self-control among middle school students [32]. This scale consists of 10 items rated on a Likert 5-point scale, where higher scores indicate lower self-control capabilities. The Cronbach’s alpha coefficient for the scale in this study was 0.81.

#### Difficulties in emotion regulation scale-short form

Emotion regulation difficulties were measured using the Difficulties in Emotion Regulation Scale-Short Form (DERS-16) [33]. This scale consists of 16 items and is rated using the Likert 5-point scale (1 = almost never, 5 = almost always). The higher the score, the greater the difficulty in regulating emotions. Previous research has demonstrated the suitability and good reliability and validity of this scale for the middle school student population [34]. The Cronbach’s alpha coefficient for the scale in this study was 0.94.

### Procedure

Prior to the initiation of the study, informed consent forms were required to be signed by all participants and their guardians. The students completed the aforementioned suite of psychological assessment tools in a quiet and minimally disruptive computer lab setting. The questionnaires were distributed via an online survey platform, Questionnaire Star (www.wjx.cn), with students responding to the items on computers. To ensure the accuracy and reliability of the data collected, at least two researchers were present in each computer lab to provide necessary guidance to the students before they began answering the questionnaires and to clarify any questions or concerns during the process.

### Data analysis

All analyses in this study were conducted using SPSS software, version 20. The chained mediation model was analyzed using Model 6 of the PROCESS macro with 5,000 bootstrap samples [35, 36]. Chained mediation involves a sequence of mediation steps where one mediator variable affects another in a chain-like process, ultimately linking the independent variable to the dependent variable through multiple mediators. The procedure involves the following steps: (a) Specify the independent variable (sleep quality), dependent variable (emotion regulating difficulties), and the mediator variables (daytime dysfunction, social exclusion, and self-control); (b) Use the PROCESS macro to define the model number (Model 6) that supports chained mediation; (c) Set the number of bootstrap samples (e.g., 5,000) to estimate the indirect effects with confidence intervals. (d) Run the analysis to obtain the path coefficients and significance levels for each mediation step. A 95% confidence interval (CI) for the mediation coefficients that does not include zero indicates a significant mediation effect [35]. Due to significant differences in demographic variables such as age and gender across variables, we have included them as covariates in the analysis to control for their potential confounding effects. To control the false discovery rate in multiple testing, we applied the Benjamini-Hochberg procedure to adjust all *p*-values, using an FDR level of 0.05 [37].

To assess the presence of common method bias, Harman’s single-factor test was conducted. The analysis revealed that four factors had eigenvalues greater than 1, with the first factor explaining 28.47% of the variance, which is below the critical threshold of 40% [38, 39]. This result suggests that common method bias is not a significant concern in this study. Furthermore, to mitigate the risk of multicollinearity, the primary variables were mean-centered. Centering involves subtracting the mean score of a variable from each individual score, thereby aligning the mean of the transformed variable with zero. This process can help to clarify the interpretation of interaction terms in regression analyses and reduce multicollinearity among predictors, ensuring the stability and reliability of the regression coefficients.

According to G*Power analysis, the present study possesses a statistical power of 0.91 to detect small effect sizes (*f*^*2*^ = 0.02).

## Results

### An analysis of the correlation among gender, grade, late sleeping habits, relationship with parents, sleep quality, daytime dysfunction, social exclusion, self-control, and emotion regulation difficulties

Table 1 presents the correlation coefficients among Gender, Grade, Late Sleeping Habits, Relationship with Parents, Sleep Quality, Daytime Dysfunction, Social Exclusion, Self-Control, and Emotion Regulation Difficulties.

**Table 1:** Descriptive statistics, correlation, and reliabilities for all variables

	1	2	3	4	5	6	7	8	9	10
1. Gender	1.00									
2. Grade Level	0.06	1.00								
3. Relationship with Father	− 0.17^**^	− 0.07^*^	1.00							
4. Relationship with Mother	0.03	− 0.07^*^	0.46^**^	1.00						
5. Late Sleeping Habits	0.11^**^	0.51^**^	− 0.17^**^	− 0.15^**^	1.00					
6. Sleep Quality	0.02	0.27^**^	− 0.19^**^	− 0.19^**^	0.51^**^	1.00				
7. Daytime Dysfunction	0.14^**^	0.42^**^	− 0.25^**^	− 0.22^**^	0.53^**^	0.57^**^	1.00			
8. Social Exclusion	− 0.03	− 0.06	− 0.26^**^	− 0.25^**^	0.13^**^	0.19^**^	0.19^**^	1.00		
9. Self-Control	0.17^**^	0.19^**^	− 0.32^**^	− 0.25^**^	0.26^**^	0.29^**^	0.43^**^	0.31^**^	1.00	
10. Difficulty in Emotion Regulation	0.16^**^	0.09^**^	− 0.30^**^	− 0.26^**^	0.27^**^	0.30^**^	0.42^**^	0.33^**^	0.54^**^	1.00
M	-	-	5.03	5.94	2.35	2.44	5.34	30.31	29.67	35.62
SD	-	-	1.62	1.33	0.85	0.85	1.83	6.37	7.27	13.91

Note. * *p* < .05, ** *p* < .01, *** *p* < .001

A simple regression analysis was conducted with emotion regulation difficulties as the dependent variable and gender, grade, late bedtime, relationship with father, and relationship with mother as covariates. Sleep quality was included as the independent variable. The results revealed a significant effect of sleep quality on emotion regulation difficulties (*β* = 3.10, *SE* = 0.61, *p* < .001, *R*^2^ = 0.19), indicating that poorer sleep quality is associated with greater difficulties in emotion regulation.

### The effect of sleep quality on emotion regulation difficulties: the chain mediating role of daytime dysfunction, social exclusion and self-control

**Fig. 2:**
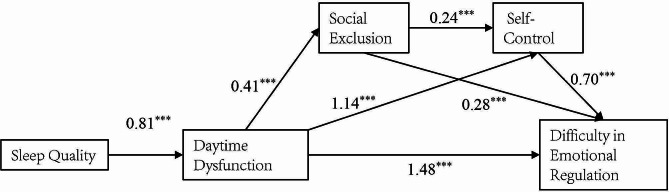
Model diagram of chain mediation effect

The analysis of the chained mediation effects, delineated the pathway from independent variables to Emotion Regulation Difficulties. The analysis demonstrated that Gender (*β* = 0.31, SE = 0.10, *p* < .01), Grade (*β* = 0.31, SE = 0.05, *p* < .001), Late Sleeping Habits (*β* = 0.46, SE = 0.07, *p* < .001), and Sleep Quality (*β* = 0.81, SE = 0.07, *p* < .001) significantly and positively influenced Daytime Dysfunction. Poorer sleep quality is associated with higher Daytime Dysfunction. Late Sleeping Habits were further associated with a significant and positive impact on Social Exclusion (*β* = 0.65, SE = 0.33, *p* < .05), as was Daytime Dysfunction (*β* = 0.41, SE = 0.15, *p* < .01). Conversely, Grade showed a significant and negative impact on Social Exclusion (*β* = -1.08, SE = 0.22, *p* < .001). In terms of Self-Control, Gender (*β* = 1.70, SE = 0.45, *p* < .001) significantly positively influenced this variable, and both Daytime Dysfunction (*β* = 1.14, SE = 0.16, *p* < .001) and Social Exclusion (*β* = 0.24, SE = 0.04, *p* < .001) emerged as significant predictors. The culmination of these pathways indicated that Daytime Dysfunction (*β* = 1.48, SE = 0.30, *p* < .001), Social Exclusion (*β* = 0.28, SE = 0.07, *p* < .001), and Self-Control (*β* = 0.70, SE = 0.06, *p* < .001) significantly and positively impacted Emotion Regulation Difficulties. Additionally, Grade had a significant and negative effect on Emotion Regulation Difficulties (*β* = -1.21, SE = 0.42, *p* < .01). Each of these pathways and their respective coefficients are detailed in Table 2; Fig. 2.

**Table 2:** Summary of the chain-mediated model of sleep quality and emotional regulation difficulties

	Emotion Regulation Difficulties	Daytime Dysfunction	Social Exclusion	Self control	Emotion Regulation Difficulties
	β (SE)	β (SE)	β (SE)	β (SE)	β (SE)
Gender	3.39 ^***^ (0.91)	0.31 ^**^ (0.10)	-0.92 ^*^ (0.44)	1.70 ^***^ (0.45)	1.85 ^*^ (0.81)
Grade	-0.66 (0.46)	0.31 ^***^ (0.05)	-1.08 ^***^ (0.22)	0.38 (0.23)	-1.21 ^**^ (0.42)
Late Sleeping Habits	2.22 ^***^ (0.67)	0.46 ^***^ (0.07)	0.65 ^*^ (0.33)	-0.17 (0.34)	0.92 (0.60)
Relationship with Father	-1.44 ^***^ (0.32)	-0.10 ^**^ (0.03)	-0.66 ^***^ (0.15)	-0.62 ^***^ (0.16)	-0.47 (0.28)
Relationship with Mother	-1.38 ^***^ (0.38)	-0.09 ^*^ (0.04)	-0.61 ^***^ (0.18)	-0.37 (0.19)	-0.61 (0.34)
Sleep Quality	3.10 ^***^ (0.61)	0.81 ^***^ (0.07)	0.55 (0.32)	0.37 (0.33)	0.59 (0.59)
Daytime Dysfunction			0.41 ^**^ (0.15)	1.14 ^***^ (0.16)	1.48^***^(0.30)
Social Exclusion				0.24 ^***^ (0.04)	0.28^***^(0.07)
Self-control					0.70^***^(0.06)
*R* ^*2*^	0.19	0.45	0.14	0.29	0.38

Note. * *p* < .05, ** *p* < 0 0.01, *** *p* < .001

### The mediating effects of various pathways through which sleep quality impacts difficulties in emotional regulation

Table 3 summarizes the mediating effects of various pathways through which sleep quality impacts difficulties in emotional regulation.

**Table 3:** Results of mediation effect test in the chain mediation model

	Estimate	*S.E.*	*Z*	Boot_95_CI
Sleep Quality → Daytime Dysfunction → Difficulty in Emotional Regulation	1.20	0.27	4.45	[0.70, 1.76]
Sleep Quality → Social Exclusion → Difficulty in Emotional Regulation	0.15	0.10	1.47	[-0.03, 0.37]
Sleep Quality → Self-Control → Difficulty in Emotional Regulation	0.26	0.24	1.08	[-0.21, 0.70]
Sleep Quality → Daytime Dysfunction → Social Exclusion → Difficulty in Emotional Regulation	0.09	0.05	1.94	[0.01, 0.20]
Sleep Quality → Daytime Dysfunction → Self-Control → Difficulty in Emotional Regulation	0.65	0.13	5.05	[0.42, 0.91]
Sleep Quality → Social Exclusion → Self-Control → Difficulty in Emotional Regulation	0.09	0.06	1.56	[-0.01, 0.21]
Sleep Quality → Daytime Dysfunction → Social Exclusion → Self-Control → Difficulty in Emotional Regulation	0.06	0.03	2.13	[0.01, 0.11]
Direct Effect	0.59	0.59	1.00	[-0.53, 1.81]
Total Effect	3.10	0.62	5.04	[1.89, 4.32]

Testing the direct effect between sleep quality and emotional regulation difficulties. The direct pathway from sleep quality to emotional regulation difficulties was not significant, with an estimate of 0.59 (*SE* = 0.59, Z = 1.00, 95% *CI* [-0.53, 1.81]). This suggests that sleep quality’s influence on emotional regulation is predominantly indirect. Significant indirect effects were found through several mediating paths.

Mediation between sleep quality, daytime dysfunction and emotional regulation difficulties. The pathway through daytime dysfunction was substantial, with an estimate of 1.20 (*SE* = 0.27, *Z* = 4.45, 95% *CI* [0.70, 1.76]), underscoring daytime dysfunction’s pivotal role in the sleep quality-emotional regulation nexus.

Mediation between sleep quality, social exclusion and emotional regulation difficulties. The mediating role of social exclusion was marginal and not statistically significant, with an estimate of 0.15 (*SE* = 0.10, *Z* = 1.47, 95% *CI* [-0.03, 0.37]).

Testing the mediation role of self-control between sleep quality and emotional regulation difficulties. The influence of self-control as a mediator also did not reach significance, with an estimate of 0.26 (*SE* = 0.24, *Z* = 1.08, 95% *CI* [-0.21, 0.70]).

The chained mediation role of daytime dysfunction and social exclusion between sleep quality and emotional regulation difficulties. Among more complex mediating pathways, the combined effect of daytime dysfunction and social exclusion was significant, with an estimate of 0.09 (*SE* = 0.05, *Z* = 1.94, 95% *CI* [0.01, 0.20]).

The chained mediation role of daytime dysfunction and self-control between sleep quality and emotional regulation difficulties. The pathway through daytime dysfunction and self-control was notably significant, yielding an estimate of 0.65 (*SE* = 0.13, *Z* = 5.05, 95% *CI* [0.42, 0.91]), indicating a strong mediating effect.

The chained mediation role of social exclusion and self-control between sleep quality and emotional regulation difficulties. The mediating effect through social exclusion and self-control as mediators was not significant. However, the comprehensive pathway encompassing daytime dysfunction, social exclusion, and self-control presented a significant mediating effect, with an estimate of 0.06 (*SE* = 0.03, *Z* = 2.13, 95% *CI* [0.01, 0.11]).

*P*-values adjusted using the Benjamini-Hochberg procedure did not significantly alter our findings, supporting the robustness of our results.

The comprehensive analysis elucidates the nuanced and interconnected pathways through which sleep quality can influence emotional regulation, highlighting the importance of targeting multiple mediatory mechanisms in interventions designed to improve emotional regulation by enhancing sleep quality.

## Discussion

This study delved into how sleep quality impacts difficulties in emotional regulation through mediators such as daytime dysfunction, social exclusion, and self-control. The findings indicate that the direct effect of sleep quality on emotional regulation difficulties is not significant. However, its influence through several mediating pathways is significant. Specifically, there is a significant mediating effect of sleep quality through daytime dysfunction on emotional regulation difficulties, highlighting the pivotal role of daytime dysfunction in the connection between sleep and emotions. Additionally, sleep quality impacts emotional regulation through social exclusion, suggesting a bridging role of social interactions between sleep and emotions. Although the effect of sleep quality through self-control on emotional regulation difficulties is not significant, significance emerges in more complex mediating pathways. The combined impact of sleep quality through daytime dysfunction and social exclusion on emotional regulation difficulties, as well as through the comprehensive pathway involving daytime dysfunction, social exclusion, and self-control, is significant, revealing the integrated role of these factors in emotional regulation. These findings underscore the importance of improving sleep quality for enhancing emotional health and hint at the critical roles of daytime dysfunction, social interactions, and individual self-control in this process.

This study demonstrated that poor sleep quality significantly and positively affects daytime dysfunction. Previous research has highlighted that poor sleep quality can lead to mental disorders and physical health issues [18]. Insufficient or poor-quality sleep may result in decreased attention, delayed response times, and emotional fluctuations, thereby impacting daytime work and learning efficiency [40]. Furthermore, the connection between daytime dysfunction and difficulties in emotional regulation was emphasized in this study. Poor sleep quality affects daytime dysfunction not only directly but also indirectly by influencing individuals’ daytime dysfunction to affect their emotional regulation. This finding extends the research by Kayaba et al. (2020), who observed that sleep problems affect students’ daytime dysfunction, including emotional fluctuations and social interactions. Our study further unveils the links between daytime dysfunction, social exclusion, and self-control. Daytime dysfunction may lead to an increased sense of social exclusion among adolescents in social settings, which in turn impacts their self-control capabilities. This intricate interplay underscores that daytime dysfunction is not merely a mental health issue but also a key factor affecting adolescents’ social interactions and emotional regulation.

Our research further revealed that poor sleep quality does not directly influence social exclusion. Instead, poor sleep quality can contribute to social exclusion through daytime dysfunction. Both daytime dysfunction and social exclusion act as chained mediators in the link between sleep quality and difficulties in emotional regulation. These findings align with the research by Hawkley et al., which explored the extensive effects of loneliness on psychological and physiological health, and found that social exclusion may lead to feelings of isolation, thereby intensifying emotional regulation issues [41]. In our study, poor sleep quality was shown to exacerbate social exclusion by increasing daytime dysfunction, in turn affecting emotional regulation. This may occur because individuals with poor sleep quality often experience increased anxiety and discomfort during social interactions, leading to social exclusion [18], and consequently, difficulties in emotional regulation.

Regarding self-control, our study findings indicate that while self-control directly impacts difficulties in emotional regulation, its role as a mediator between sleep quality and emotional regulation difficulties is not significant. This suggests that sleep quality may not significantly affect self-control. This is in contrast with some prior research, which found that sleep deprivation can impair self-control [11]. These discrepancies might be attributed to differences in sample characteristics, sleep quality assessments, or methods of measuring self-control. However, our study did find that sleep quality can influence self-control through daytime dysfunction, subsequently affecting difficulties in emotional regulation. In other words, sleep quality may be indirectly associated with difficulties in emotion regulation through chained mediating process involving daytime dysfunction and self-control.

Some of the covariates included in this study are noteworthy. The current study delineates significant gender disparities across various constructs. Specifically, females are more likely to experience difficulties in emotion regulation, daytime dysfunction, and self-control challenges compared to males. However, females may be less susceptible to feelings of social exclusion. These findings are consistent with gender-based differences documented in psychological literature, where females often report higher emotional sensitivity and reactivity, potentially leading to greater challenges in emotion regulation [42]. On the contrary, the lower incidence of social exclusion among females could be related to higher levels of social connectivity and communication skills that are, on average, more developed in females than males [43]. The effect of grade level was also significant in this study, with an increase in grade corresponding with a decrease in social exclusion and emotion regulation difficulties, but an increase in daytime dysfunction. The reduction in social exclusion and emotional difficulties with ascending grades could be attributed to the development of coping strategies and social skills as students mature [44]. Conversely, the escalation in daytime dysfunction might be due to the increasing academic demands and the associated stress that could adversely affect sleep quality and daytime alertness [45]. Furthermore, relationships with parents, particularly fathers, were inversely associated with daytime dysfunction, social exclusion, and self-control. A close relationship with one’s father was linked to stronger self-control abilities. Positive parental involvement is known to be associated with better psychological outcomes in children, including greater self-regulation and lower levels of behavioral problems [46]. This is supported by attachment theory, which posits that secure parent-child relationships provide a foundation for children’s emotional and social development [47].

This study selected participants through a random sampling of students from four different schools, including both junior and senior high schools, ensuring sample diversity and representativeness and thereby enhancing the generalizability of the findings. Moreover, the study innovatively applied a chained mediation model to explore how sleep quality, daytime dysfunction, social exclusion, and self-control jointly influence difficulties in emotion regulation. This method offers a more nuanced understanding and explanation than traditional correlational studies. Through the application of the chained mediation model, we revealed how multiple factors, such as daytime dysfunction, social exclusion, and self-control, collectively affect emotion regulation difficulties, offering new perspectives and strategies for interventions. Future research could further explore other potential mediators, such as psychological stress, cognitive functions, or family environment, to provide a more comprehensive understanding of these psychological phenomena. Based on the findings of this study, future interventions could be developed to improve sleep quality, enhance self-control, and alleviate social exclusion, with an assessment of their effectiveness in ameliorating emotional regulation difficulties.

### Limitations

This study also has limitations. Firstly, although participants were randomly sampled from four schools, all located within the same region, this may not fully represent the broader adolescent population. Future research should be conducted across a wider geographical area to improve the universal applicability of the findings. Secondly, the use of a cross-sectional design means that causality cannot be established. While the chained mediation model provides meaningful insights, the causal directions remain unclear, and some variables may have bidirectional causality. For instance, poor sleep quality may contribute to emotion regulation difficulties, but emotion regulation difficulties could also negatively impact sleep quality. Future research could employ longitudinal designs to more accurately trace and analyze changes and influences over time. Additionally, examining the bidirectional nature of these relationships is essential. Previous literature supports the idea that sleep quality and emotion regulation difficulties can influence each other reciprocally [13]. Longitudinal studies would help clarify the directionality and the complex interplay between these variables. Thirdly, the study relied on self-report measures, which might be subject to biases such as social desirability. Future studies could incorporate objective data (e.g., sleep quality monitored by wearable devices) and other-rated data (e.g., evaluations from parents, peers, and teachers) for a more comprehensive and objective analysis. Fourth, in conducting the mediation analysis with multiple mediator variables, this study is confronted with the risk of inflated Type I error rates, a consequence of multiple statistical tests. However, *p*-values adjusted using the Benjamini-Hochberg procedure did not significantly alter our findings, supporting the robustness of our results. Lastly, despite the use of validated measurement tools, the assessment of certain variables could be more comprehensive. For instance, to reduce the burden on students, only two dimensions from the Pittsburgh Sleep Quality Index were selected to assess sleep quality and daytime dysfunction.

## Conclusion

In summary, our findings indicate that poor sleep quality significantly contributes to increased daytime dysfunction in adolescents, which in turn affects social exclusion, self-control abilities and emotion regulation difficulties. Social exclusion mediates the relationship between sleep quality and emotion regulation difficulties. This study offers insights for the development of effective interventions aimed at improving sleep and psychological well-being in adolescents.

## Data Availability

Data available on request from the authors.
